# Immunocyte Populations Observed from Birth to Weaning in Blood, Spleen and Mesenteric Lymph Nodes of Piglets

**DOI:** 10.3390/ani12111445

**Published:** 2022-06-03

**Authors:** Tomoko Harayama, Takamitsu Tsukahara, Kikuto Fukuta, Machi Oda, Ryo Inoue

**Affiliations:** 1 Laboratory of Animal Science, Division of Applied Life Sciences, Graduate School of Life and Environmental Sciences, Kyoto Prefectural University, Kyoto 606-8522, Japan; reeforth73finag@aol.com (T.H.); ryo.inoue@setsunan.ac.jp (R.I.); 2 Kyoto Institute of Nutrition & Pathology, Kyoto 610-0231, Japan; 3 Technical Center, Toyohashi Feed Mills, Aichi 441-1346, Japan; k-hukuta@toyohashi-shiryo.co.jp; 4 Laboratory of Animal Science, Department of Applied Biological Sciences, Faculty of Agriculture, Setsunan University, Hirakata 573-0101, Japan; machi.oda@edu.setsunan.ac.jp

**Keywords:** immunocyte population, neonatal piglet, mesenteric lymph node cell, peripheral blood mononuclear cell, splenocyte

## Abstract

**Simple Summary:**

Understanding the immunocyte development in neonatal piglets is crucial because susceptibility to pathogen infections and efficacy of vaccination highly depend on the immune status of the piglet. However, the immunocyte information on the period between birth and weaning remains scarce, especially that on immune tissues other than blood, such as mesenteric lymph nodes (MLN) and spleens. Our results indicated that the percentage of innate immunity cells such as myeloid cells, most of them possibly being monocytes, declined from the first day after birth in blood, MLN and spleens. By contrast, other innate immunocytes such as natural killer cells and adaptive immunocytes such as T and B cells were produced while the piglets still suckled in the three organs. Weaning did not seemingly affect the dynamics of the studied immunocytes in a drastic manner, at least during the 14 days post weaning. We foresee that the information from the present study warrants the design of better programs for the administration of vaccines and/or innate immunity stimulators to suckling and weaned piglets.

**Abstract:**

Susceptibility to pathogen infections and efficacy of vaccination highly depend on the immune status of the piglet. Here, we measured immunocytes in piglets from birth to weaning to elucidate how immunocyte populations change during development and are affected by weaning. Crossbred piglets were used. Suckling piglets were euthanized at 1, 7, 14, 21, 28 or 35 days old (3~4 piglets at each time point). In addition, seven piglets were weaned at 21 days old, with four being euthanized at 28 days old and the remaining at 35 days old. Piglet carcasses were dissected, and blood, mesenteric lymph nodes (MLN) and spleen were sampled. In total, seven antibodies were used to stain the immunocyte population. Dynamics of myeloid (CD3^–^SWC3^+^CD16^+^), natural killer (NK; CD3^–^SWC3^–^CD16^+^), killer T (CD3^+^CD8^+^), helper T (CD3^+^CD4^+^) and B (CD3^–^CD21^+^) cells were analyzed. Percentage of innate immunity cells such as myeloid cells declined (*p* < 0.05) from the first day after birth. In contrast, percentage of NK cells increased in piglets while they were still suckling. Killer T, helper T, and B cell populations increased around 2~3 weeks after birth. No significant differences in the populations of the evaluated cell types were observed between suckling and weaned piglets at least for 14 days post weaning.

## 1. Introduction

In the swine industry, antimicrobial treatment is a frequent technique to prevent and/or cure pathogenic infections [1]. Nonetheless, although bactericidal and bacteriostatic compounds are usually effective against pathogenic bacteria, antimicrobials also adversely affect the commensal microbiota in the gut of pigs [1,2]. Recently, the National Veterinary Assay Laboratory in Japan [3] issued a guideline for the “Prudent Use of Veterinary Antimicrobial Medicines”. To protect animals and the pig industry, this guideline requests the development of alternative immune intervention materials such as probiotics [4,5,6] and vaccines [7,8].

In pig production in particular, vaccination is expected to directly prevent the clinical signs of diseases [7]. Due to the many types of vaccines administered to piglets, vaccination programs are considered very important measures to prevent diseases from spreading within and across pig farms [7,9]. However, the efficacy of vaccines depend on the immune status of piglets [7,9]. This is also the case for probiotics and prebiotics with immunomodulatory effects. However, the information on the period between birth and weaning remains scarce, especially that on immune tissues other than blood, such as mesenteric lymph nodes (MLN) and spleens.

Every year, a number of highly infectious diseases cause considerable economic damage to the pig industry [10]. Under the current breeding systems of commercial farms, weaning is one of the most stressful events for growing piglets [11] and those considered still underdeveloped [12]. Indeed, due to changes in feed and environment, and encounters with unfamiliar animals, etc. [11], weaning, in particular, makes piglets susceptible to numerous antigens [13,14] and potential pathogens [15]. Hence, knowledge on how weaning impacts the immunocyte population is of particular importance to manage weanling piglets.

In the present work, we aimed to determine the dynamics of various immunocytes in blood and secondary immune tissues such as MLN and spleen in both suckling and weaned piglets. To that effect, we measured the populations of adaptive immune cells such as helper T, killer T and B cells, and those of innate immune cells such as myeloid and natural killer (NK) cells.

## 2. Materials and Methods

### 2.1. Animals and Cell Collection

The present study was carried out in accordance with the guidelines for animal studies issued by the Experimental Animal Committee of Kyoto Prefectural University and its protocol approved by this Committee (Approval number: KPU240410).

Piglets were crossbred (Landrace × Large white × Duroc) and raised at the Technical Center of the Toyohashi Feed Mills (Shinshiro, Aichi, Japan). The technical Center is an experimental farm where the sanitary conditions are kept at the highest level possible. Following a screening of the piglets for the possible presence of pathogens, only porcine circovirus type II was detected, while the results for others such as porcine reproductive and respiratory syndrome virus and pathogenic *Escherichia coli* were negative. Certain body parts of the dissected piglets used for the present study were the same as those used in a previously reported work [13].

For the present work, piglets were obtained from different herds, due to the fact that the number of animals available was limited. Thus, 30 piglets were obtained from litters of four different Landrace × Large white sows. Based on body conditions at the time of group allocation, all selected piglets were considered suitable for sampling. For the present study, parts of piglets were also selected and grouped as previously described [13]. Twenty-three suckling piglets were selected and euthanized at each of the following ages: 1 day old (S1D; *n* = 4), 7 days old (S7D; *n* = 4), 14 days old (S14D; *n* = 4), 21 days old (S21D; *n* = 4), 28 days old (S28D; *n* = 4) and 35 days old (S35D; *n* = 3). In addition, seven piglets were selected as the weaning group and weaned at 21 days of age. These piglets were euthanized at 28 days (21W28D; *n* = 4) and 35 days (21W35D; *n* = 3) of age. While weaning piglets were given a typical commercial diet for weanlings (JustOne Sprout; Toyohashi Feed Mills, Aichi, Japan), suckling piglets had only their mothers’ milk. During the suckling period, piglets were observed to be less stressful, perhaps because some littermates were gradually removed from the sows for dissection. After weaning, all weaned piglets were kept in a mesh-floored pen (W 1.800 × D 2.100 m) in the weaning room. The average body weight of each piglet group at farrowing and slaughter were previously reported [13]. While none were vaccinated, all animals used in the present study received were healthy, with no evident infectious symptoms.

Piglets were anesthetized by an intraperitoneal injection of sodium pentobarbital (Somnopentyl; Kyoritsu, Tokyo, Japan). The abdomens of the piglet carcasses were incised, and prior to exsanguination, blood was collected from the abdominal veins and into heparinized tubes (Venoject II vacuum blood collection tubes, Terumo Medical Corp., Tokyo, Japan). Next, spleen and MLN were resected from the carcasses. Splenocytes and MLN cells were retrieved using a previously described procedure [16]. Residual pellets of splenocyte or MLN cells were resuspended in appropriate volume (5 × 10^6^ cells/mL) of 0.5% bovine serum albumin (BSA)-supplemented phosphate-buffered saline (PBS; 137 mmol/L NaCl, 2.68 mmol/L KCl, 10 mmol/L Na_2_HPO_4_ and 2 mmol/L KH_2_PO_4_ at pH 7.4).standing

Four mL of heparinized blood was transferred to 15-mL centrifuge tubes and gently mixed with 8 mL of a red blood cell lysis buffer (0.5 mol/L NH_4_Cl, 10 mmol/L KHCO_3_ and 0.1 nmol/L Na_2_EDTA at pH 7.2). After 5 min of room temperature (RT) incubation, the mixture was centrifuged (400× *g*, 5 min, RT) and the supernatant removed. All residues were removed by washing them with PBS. After further centrifugation (400× *g*, 5 min, RT), cell pellets were suspended in 4 mL of the red blood cell lysis buffer and the suspensions were vortexed. Cells were incubated again and then washed with PBS. The new suspensions were centrifuged (400× *g*, 5 min, RT) again, and the residual pellets were retrieved and resuspended in appropriate volume (5 × 10^6^ cells/mL) of 0.5% BSA-supplemented PBS. These cell suspensions were considered as peripheral blood mononuclear cells (PBMC).

### 2.2. Immunocyte Population Analysis by Flow Cytometry

Immunocyte populations were determined by staining with membrane markers, as previously reported by Piriou-Guzylack and Salmon [17] and Summerfield and McCullough [18].

MLN cells, splenocytes and PBMCs were divided in three aliquots. To measure the populations of myeloid and natural killer (NK) cells, one aliquot (200 µL; 1 × 10^6^ cells) of each cell suspension was used for immunocyte population analysis and had 0.2 µL of anti-SWC3 (mouse IgG1 clone DH59B; 1:1000; Veterinary Medical Research & Development (VMRD), Pullman, WA, USA) added. The suspensions were gently mixed and transferred to screw-capped tubes. These mixtures and all subsequent mixtures were conducted in screw-capped tubes and incubated at room temperature for 30 min in a dark place. After incubation, the mixtures were centrifuged (500× *g*, 3 min, 4 °C). After centrifugation, the supernatants were removed and washed with 500 µL of 0.5% BSA-supplemented PBS. The new mixtures were centrifuged as described above, and the residual pellets were resuspended in 500 µL of 0.5% BSA-supplemented PBS. Two µL of secondary antibody against mouse IgG1 (PerCP conjugated goat polyclonal; 1:250; Santa Cruz biotechnology, Dallas, TX, USA) was then added to the suspensions, and these were incubated. Afterwards, cell suspensions were washed with 0.5% BSA-supplemented PBS, and cell pellets were resuspended in 500 µL of 0.5% BSA-supplemented PBS. Afterwards, 0.2 µL of PE conjugated anti-porcine CD3 (clone PPT3; 1:2,500; Beckman Coulter, Brea, CA, USA) and FITC-conjugated 0.2 µL of anti-porcine CD16 (clone G7; 1:2,500; Nippon Becton, Dickinson and Company, Tokyo, Japan) were added and the mixtures were incubated. The cell suspensions were then washed with 0.5% BSA-supplemented PBS. Cell pellets were resuspended in 500 µL of 1% paraformaldehyde, and these cell suspensions were analyzed by flow cytometry (FACS calibur; Nippon Becton, Dickinson and Company, Tokyo, Japan). The cell populations of CD3^–^CD16^+^SWC3^+^, and CD3^–^CD16^+^SWC3^–^ were obtained, which permitted the identification of myeloid cells and NK cells, respectively.

Additional aliquots of the MLN cell, splenocyte and PBMC suspensions were used to measure killer T and B cells. An aliquot (0.4 µL) of anti-porcine CD21 (mouse IgG1 clone BB6-11C9; 1:500; VMRD) was added to the cell suspensions and the mixtures were incubated. The mixtures were centrifuged (500× *g*, 3 min, 4 °C), and the cell pellets were retrieved and washed with 0.5% BSA-supplemented PBS. These cell suspensions were centrifuged again (500× *g*, 3 min, 4 °C), and the retrieved cell pellets were resuspended in 500 µL of 0.5% BSA-supplemented PBS. Three µL of a secondary antibody against mouse IgG1 (PerCP conjugated goat polyclonal; 1:167; Santa Cruz biotechnology, Dallas, TX, USA) was added to the cell suspensions and the mixtures were incubated again. Next, cell suspensions were washed with 0.5% BSA supplemented PBS. Cell pellets were then resuspended in 500 µL of 0.5% BSA-supplemented PBS, along with 0.2 µL of PE conjugated anti-CD3 (clone PPT3; 1:2500; Beckman Coulter, Brea, CA, USA) and FITC conjugated 0.4 µL of anti-CD8 alpha (clone 76-2-11; 1:1250; Abcam, Cambridge, UK). These mixtures were incubated again. After incubation, cell suspensions were washed with 0.5% BSA-supplemented PBS. Finally, cell pellets were resuspended in 500 µL of 1% paraformaldehyde, and these cell suspensions were analyzed by flow cytometry. The cell populations of CD3^+^CD8^+^CD21^–^ and CD3^–^CD8^–^CD21^+^ cells were obtained, which permitted the identification of killer T and B cells, respectively.

The remaining aliquots of the MLN cell, splenocyte and PBMC suspensions were analyzed for helper T cells. Aliquots (0.2 µL each) of PE conjugated anti-porcine CD3 (clone PPT3; 1:1000; Beckman Coulter) and FITC conjugated anti-porcine CD4 (clone 74-12-4; 1:1000; Abcam) were mixed and the mixtures were incubated again, after which they were washed with 0.5% BSA-supplemented PBS. After centrifugation, the retrieved cell pellets were resuspended in 500 µL of 1% paraformaldehyde, and these cell suspensions were analyzed by flow cytometry. The cell populations of CD3^+^CD4^+^ were obtained, which permitted the identification of helper T cells.

In the present study, we applied secondary antibody staining during the staining procedure to use non-labeled antibody (Figure S1). We corroborated in advance that this staining procedure did not affect the staining pattern. In other words, the staining with multiple antibodies produced results comparable with the staining with every single antibody. As an example, a comparison of the staining patterns using multiple staining (anti-CD3, CD16, SWC3) and every individual antibody is shown in Figure S2.

### 2.3. Concentration of IL-12 in Serum

Serum was collected from the remaining peripheral blood by centrifugation (1750× *g*, 10 min, 4 °C). The concentration of IL-12p40 in the serum was measured with a commercial ELISA kit (Porcine IL-12/IL-23 p40 DuoSet ELISA; R&D Systems, Minneapolis, MN, USA).

### 2.4. Statistical Analyses

Differences between variables at slaughter points, namely S1D, S7D, S14D, S21D, S28D, and S35D (suckling piglets), and 21W28D and 21W35D (weaned piglets) were analyzed by one-way analysis of variance (ANOVA). When significant differences were detected, Tukey–Kramer post hoc comparisons were used instead. Differences between the means were considered significant when *p* < 0.05. All data were analyzed using STATCEL (OMS, Saitama, Japan), an add-in application for Microsoft Excel© (Seattle, WA, USA).

## 3. Results

### 3.1. Percentages of Myeloid Cells (CD3^–^CD16^+^SWC3^+^) in Blood and Immune Tissue Samples of Piglets

**Figure 1:**
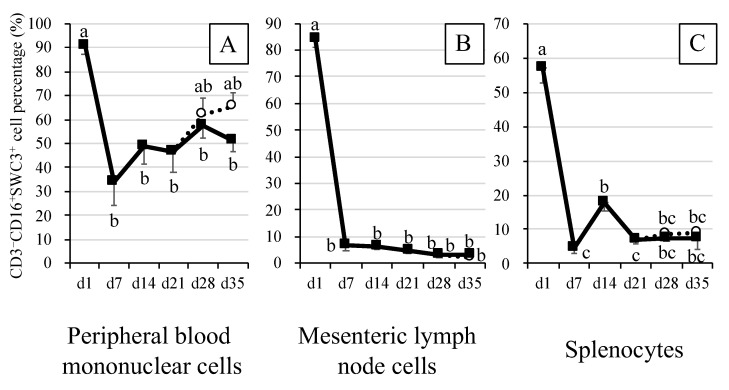
Myeloid cell population in peripheral blood mononuclear cells, mesenteric lymph node (MLN) cells and splenocytes of piglets. CD3^–^CD16^+^SWC3^+^ cells were considered as myeloid cells. Closed squares indicate the mean values for suckling piglets. Open circles indicate the mean values for piglets weaned at 21 days of age. The error bars represent the standard errors. Different superscripts indicate significant differences between groups. Panel (**A**), myeloid cell population in peripheral blood mononuclear cells. Panel (**B**), myeloid cell population in MLN cells. Panel (**C**), myeloid cell population in splenocytes.

In blood, from S7D to S35D, the percentages of myeloid cells were lower (*p* < 0.05) when compared with that detected on S1D (Figure 1A). When compared, the percentages of myeloid cells tended to be higher in weaned piglets (21W28D, 21W35D) than in suckling piglets (S28D, S35D), but the differences were non-significant.

In MLN, on S7D, S14D, S21D, S28D, S35D, 21W28D and 21W35D, the percentages of myeloid cells were significantly (*p* < 0.05) lower than that detected on S1D (Figure 1B).

In splenocytes, the percentages of myeloid cells varied depending on the experimental day for both suckling and weaned piglets. For example, compared with that detected on S1D, the percentage of myeloid cells on S7D was the lowest (*p* < 0.05) recorded in the experimental period (Figure 1C). However, the percentage of myeloid cells was found to have increased more than 10-fold (*p* < 0.05) on S14D, before lowering again on S21D to a percentage similar to that observed on S7D. Nonetheless, no differences between percentages of myeloid cells were observed on S28D, S35D, 21W28D and 21W35D, and on S7D, S14D and S21D.

### 3.2. Percentages of Natural Killer Cells (CD3^–^CD16^+^SWC3^–^) in Blood and Immune Tissue Samples of Piglets

**Figure 2:**
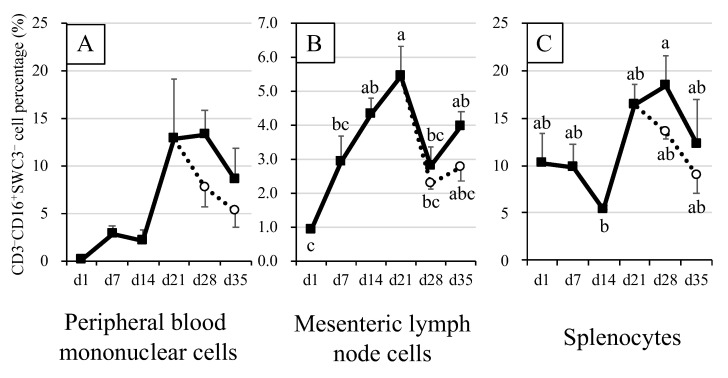
Natural killer cell population in peripheral blood mononuclear cells, mesenteric lymph node (MLN) cells and splenocytes of piglets. CD3^–^CD16^+^SWC3^–^ cells were considered as natural killer (NK) cells. Closed squares indicate the mean values for suckling piglets. Open circles indicate the mean values for piglets weaned at 21 days of age. The error bars represent the standard errors. Different superscripts indicate significant differences between groups. Panel (**A**), NK cell population in peripheral blood mononuclear cell. Panel (**B**), NK cell population in MLN cells. Panel (**C**), NK cell population in splenocytes.

On S28D, although they had a tendency to increase, no significant differences were found between the NK cell percentages in blood of both suckling and weaned piglets at different slaughter days (Figure 2A). Similarly, there was a tendency to decrease without significant differences in the percentage of NK cells of weaned piglets on 21W28D and 21W35D, when compared with those of suckling piglets (S28D, S35D).

The percentages of NK cells in the MLN cells of suckling piglets showed significant (*p* < 0.05), gradual increases from S1D through S21D (Figure 2B). However, on S28D, the NK cell percentage decreased (*p* < 0.05) before showing a tendency to increase on S35D. A similar, significant (*p* < 0.05) trend in percentages of NK cells was observed in MLN samples of weaned piglets. However, when compared, the changes in NK cell percentages between suckling (S28D, S35D) and weaned piglets (21W28D, 21W35D) non-significantly different, being those of sucking piglets relatively higher than those of weaned piglets.

In splenocytes of both suckling and weaned piglets, the percentages of NK cells showed a trend similar to those in blood (Figure 2A,C). From S1D to S7D, the NK cell percentages changed little, but by S14D, the percentages had decreased by half (*p* < 0.05), before increasing again more than 3-fold (*p* < 0.05), on S21D-S28D. A tendency to decrease was observed on S35D, but it was not significant. When compared, the NK cell percentages tended to be lower in splenocytes of weaned piglets (21W28D, 21W35D) than in those of suckling piglets (S28D, S35D).

### 3.3. Percentages of Killer T (CD3^+^CD8^+^), Helper T (CD3^+^CD4^+^) and B (CD3^–^CD21^+^) Cells in Blood and Immune Tissue Samples of Piglets

**Figure 3:**
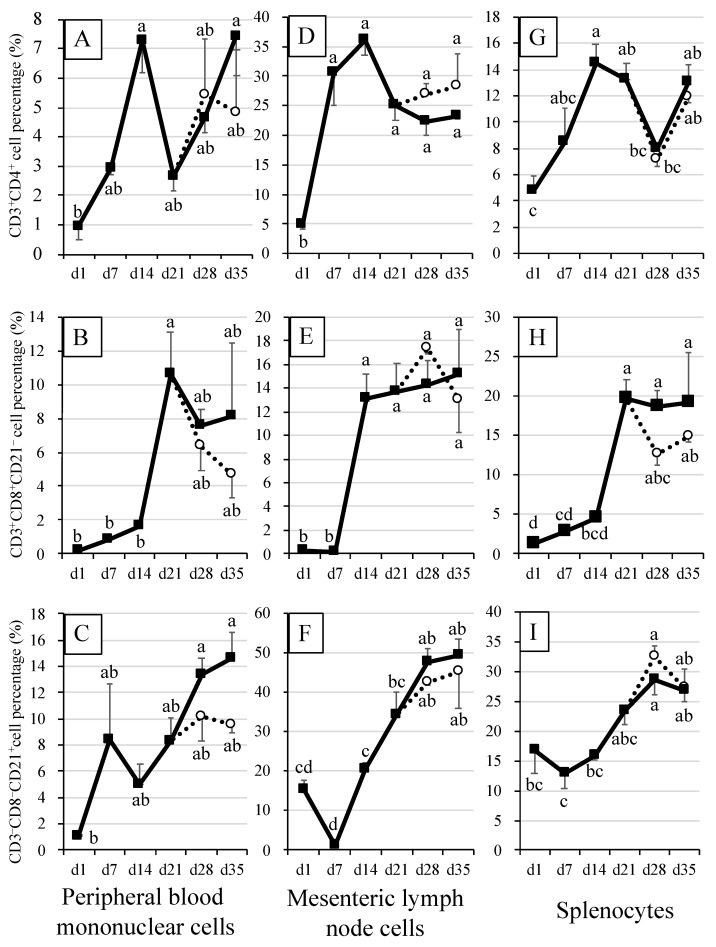
Immune cells in peripheral blood mononuclear cells (PBMC), mesenteric lymph node (MLN) cells and splenocyte of piglets and their association with adaptive immunity. CD3^+^CD4^+^ cells were considered as helper T cells, CD3^+^CD8^+^CD21^–^ cells as killer T cells, and CD3^–^CD8^–^CD21^+^ cells as B cells. Closed squares indicate the mean values for suckling piglets. Open circles indicate the mean values for piglets weaned at 21 days of age. The error bars represent the standard errors. Different superscripts indicate significant differences between groups. Panel (**A**), helper T cell population in PBMC. Panel (**B**), killer T cell population in PBMC. Panel (**C**), B cell population in PBMC. Panel (**D**), helper T cell population in MLN cells. Panel (**E**), killer T cell population in MLN cells. Panel (**F**), B cell population in MLN cells. Panel (**G**), helper T cell population in splenocytes. Panel (**H**), killer T cell population in splenocytes. Panel (**I**), B cell population in splenocytes.

In the blood of suckling piglets, the percentage of helper T cells consistently increased (*p* < 0.05) from S1D to S14D, before showing a non-significant decrease on S21D (Figure 3A). Nonetheless, when observed on S28D and S35D, the percentages of helper T cells recovered (*p* < 0.05) to levels similar to that observed on S14D. A similar trend was observed for helper T cells in blood of weaned piglets, except that after decreasing (*p* < 0.05) on S21D, the recovery observed on 21W28D was smaller. When compared, the percentages of helper T cells tended to be lower in the blood of weaned piglets (21W35D) than those of suckling piglets (S35D) but the differences were not significant. The percentages of killer T cells changed little from S1D through S14D, before significantly (*p* < 0.05) increasing from S21D through S35D (Figure 3B). When compared, the percentages of killer T cells in the blood of weaned piglets (21W28D, 21W35D) tended to be lower than those of suckling piglets (S28D, S35D), but the differences were not significant. By contrast, the percentages of B cells in the blood of both suckling and weaned piglets showed a significantly (*p* < 0.05) increasing trend throughout the experimental period (S1D –S35D), except for that observed on S14D, when they decreased, although not to a significant level (Figure 3C). On days 28 and 35, the percentages of B cells tended to be lower in the blood of weaned piglets (21W28D, 21W35D) than those of suckling piglets (S28D, S35D), but the differences were non-significant.

In MLN of both suckling and weaned piglets, the percentages of helper T cells markedly increased (*p* < 0.05) from S1D to S14D, after which they slightly decreased, although not to significant levels (Figure 3D). On days 28 and 35, the percentages of helper T cells tended to be higher in weaned piglets (21W28D, 21W35D) than those of suckling piglets (S28D, S35D). Compared with helper T cells, similar trends were observed in the percentages of killer T and B cells in MLN of suckling piglets, except that this time, no decrease whatsoever was observed (Figure 3E,F). On days 28 and 35, the percentages of killer T cells and B cells in MLN of weaned piglets (21W28D, 21W35D) were similar to those of suckling piglets (S28D, S35D) (Figure 3E,F).

In splenocytes, the percentages of helper T cells varied across time in the same manner for both suckling and weaned piglets. For example, helper T cells significantly (*p* < 0.05) increased from S1D to S14D, before significantly (*p* < 0.05) decreasing on S28D (Figure 3G). On S35D, the percentages of helper T cells increased again, although not to significant levels. Moreover, in splenocytes of suckling piglets, the percentages of killer T cells significantly (*p* < 0.05) increased from S1D to S21D, after which they plateaued (Figure 3H). Weaning seemed to affect the killer T cell percentages in MLN cells. For example, when compared on days 28 and 35, the percentages of killer T cells tended to be lower in weaned piglets (21W28D, 21W35D) than in suckling piglets (S28D, S35D), although the differences were non-significant. Lastly, the percentages of B cells in splenocytes showed similar trends in both suckling and weaned piglets (Figure 3I). For example, the percentages of B cells tended to decrease from S1D to S7D. Afterwards, B cells consistently increased through S28D before plateauing at 28 days old. When compared, the percentages of B cells in 28-day-old weaned piglets tended to be higher than that in 28-day-old suckling piglets, although these differences were not significant.

### 3.4. IL-12p40 Concentration in the Serum

The concentrations of IL-12p40 consistently increased from S7D to S28D, though the increase was non-significant (Figure S3). In addition, weaning did not induce significant changes in the concentrations of IL-12 p40. 

## 4. Discussion

The transitional period from suckling to weaning is a crucial window during which not only the highest rate of mortality is observed in offspring of mammals such as pigs, but also the passive immune system is temporarily replaced by a still developing adaptive immune system [19]. In the present work, we wanted to characterize the dynamics of immunocytes in blood and immune tissues during this transitional period and to discuss the possible effect of weaning on these dynamics. However, as weaning did not significantly affect the dynamics of the studied immunocytes, we chose instead to discuss the changes in immunocyte percentages observed during the suckling development.

In the present study, myeloid cells were observed in blood, spleen, and MLN of piglets. The percentages of these cells markedly decreased from S1D to S7D (Figure 1). As our ongoing work using additional antibody anti-CD14 seems to indicate that most of the myeloid cells (CD3^–^CD16^+^SWC3^+^) are CD14^+^ cells, most of them were expected to be monocytes in the present study [18]. Grierson et al., reported that in piglets of clinical swine farms, the percentages of monocytes (in relation to total PBMC) decreased from week 1 to week 3 post birth (54.5% vs. 32.7%) [20]. Likewise, Juul-Madsen et al., reported that in suckling piglets, the percentages of neutrophils (in relation to total leukocytes) decreased from week 1 to week 2 post birth [21]. While acknowledging that thorough comparisons of the data by Grierson et al. and Juul-Madsen et al. with those from the present study were complex, due to the differences between ages of the piglets, it can be inferred that decreases in the percentages of myeloid cells, considered as PBMC, could have actually occurred at the very early stages of the piglets’ lives. These changes may have stemmed from the increases in the percentages of other immunocyte such was lymphocytes, during the suckling period [21]. In addition to the percentages of PBMC, the concentrations of IL-12p40 in the sera of the piglets were also analyzed (Figure S3). IL-12 is mainly secreted by myeloid cells, namely macrophages, monocytes, and dendritic cells [22]. Furthermore, IL-12p40 plays a crucial role in the stimulation and potentially the maintenance of adaptive immune responses such as those of Th1 cells [23]. While no significant differences were observed, the concentrations of IL-12p40 and populations of myeloid cells tended to be the lowest (Figure S3) and the highest (Figure 1), respectively, in 1-day-old piglets. These results seemed to suggest that the myeloid cells in 1-day-old piglets were immature.

Conversely, the percentage of NK cells were observed to increase from S14D (Figure 2). However, unlike in the present study, work carried out elsewhere [24] did not find significant differences in NK populations in piglets of different ages. A possible explanation for the discrepancy between our results and those of Talker et al., may be that their time intervals for sampling were greater than ours (e.g., weeks vs. days). Indeed, since NK cells production can spike in a very short period of time in bone marrow and proliferate very rapidly [25], it is likely that our sampling within shorter periods of time was able to detect changes that otherwise would not have been possible if it was carried out within longer intervals. 

In pigs, B cells production starts in the bone marrow as early as on day 20 of gestation [26], after which they migrate as transitional B cells into the spleen and MLN [27]. By contrast, helper T cells are only mobilized after circulating particles are perceived as potential danger by antigen-presenting cells [28]. Helper T cells ultimately stimulate B cells to produce the required immunoglobulin types as well as to activate toxic cells such as killer T cells to fight the seemingly potential pathogen [29]. In the present work, this immunocyte cascade was clearly observed in blood, spleen and MLN of both suckling and weaned piglets (Figure 3). Indeed, an increase in the population of helper T cells was observed from S1D in blood, spleen and MLN, which was followed by production of B cells on S7D and subsequent activation of killer T cells on S14D (Figure 3). 

With respect to the differences in immunes cells in the analyzed blood samples and immune tissues, it was reported that subpopulations of immune cells in immune tissues were not necessarily the same as those observed in PBMC. Indeed, Gerner et al. [30] showed that, in 3-month-old piglets, robust populations of CD8β^+^ T cells (producers of effector cytokines such as IFN-γ and TNF-α) were found in MLN but only minimally in PBMC. A detailed characterization of immune cells subpopulations was beyond the scope of the present study; hence, we used only a limited number of antibodies in the analyses. This was the case not only of T cells but also of other immunocytes such as NK cells. In future work, the use of a larger array of antibodies could well unveil more detailed differences between immunocytes populations, both in blood and immune tissues.

It is worth noting that a great number of mononuclear cells such as B cells, monocytes, macrophages, granulocytes, helper T cells and killer T cells can be found in the colostrum of sows [31,32], and these immunocytes are transferred to the blood of neonatal piglets [33]. In the present work, a temporary increase in B cells in blood observed on S7D (Figure 3C) may have been associated with the colostrum suckled from sows by the piglets [34]. 

No manifested effect of weaning was observed on any of the evaluated cell types. Weaning is a very stressful period for piglets, who must quickly adapt to environmental, behavioral and physiological changes [11]. Thus, the results obtained in the present study were somewhat surprising. In the present work, animals from an experimental farm were used, and thus, weaning was conducted with more intensive care, which perhaps may explain the fact that weaned piglets had no evident infections and/or diarrheic symptoms. In the present study, the fact that no significant differences were observed between the cell types studied in both suckling and weaned piglets seems to imply that the effect of weaning on piglets was relatively uneventful. However, it must be mentioned that to identify the cell types, the present work only evaluated the expression, but not the functions (e.g., activation or proliferation) of surface markers. Hence, to demonstrate the impact of weaning on immunocytes, it is recommended that in future work, the functional changes in each of the cell types are studied. 

In the present study, there were several limitations that must be addressed. First, relatively low numbers (*n* = 3–4) of piglets were used at the observation points (ages) of the study. Previous work [35,36,37] looking into the immune development in organs of neonatal piglets, reportedly used a similar number of piglets (*n* = 4) (it conducted a dissection study). In contrast, other studies used more piglets to determine to carry out similar work [20,21], but analyzed only PBMC (no dissection studies were carried out). We duly acknowledge that a greater number of dissected piglets would most likely generate more robust results and this point will be taken into consideration for future work. Second, the piglets used in the present work were unvaccinated and raised in highly aseptic conditions. As vaccination [7] and pathogenic infections [38] induce particular immune responses in animals, in the present study, we wanted to observe a more “natural” immune development in neonatal piglets. Nonetheless, vaccination is nowadays a common strategy to prevent diseases [7] and certain pathogenic infection (especially viral diseases) worldwide. Therefore, it can be argued that our results did not represent the general health profiles of piglets observed in modern commercial farms. 

## 5. Conclusions

In conclusion, we found that in piglets, the ratio of innate immunity cells such as myeloid cells, most of them possibly being monocytes, declined from the first day after birth. By contrast, other innate immunocytes such as NK cells and adaptive immunocytes such as T and B cells were produced while the piglets still suckled. Weaning did not seemingly affect the dynamics of the studied immunocytes in a drastic manner, at least during the 14 days post weaning. Finally, adaptive immunocytes rapidly developed from 7 to 14 days of age, and as a result, antigen recognition may have started from this period onwards. To better understand the immune system status of newborn piglets, and due to biological functions of immunocytes were not initially examined, an additional study was required. The resulting data seemed to suggest that the myeloid cells in 1-day-old piglets were immature.

## Data Availability

Data are available by corresponding author.

## Supplementary Materials

The following supporting information can be downloaded at: https://www.mdpi.com/article/10.3390/ani12111445/s1, Figure S1: Flow of multiple antibody staining; Figure S2: Effect of staining with multiple antibodies on each cell population. Figure S3: Concentrations of IL-12p40 in the sera of piglets.
