# Supplementary material for: Immunocyte Populations Observed from Birth to Weaning in Blood, Spleen and Mesenteric Lymph Nodes of Piglets
Source: Animals (Basel). 2022 Jun 3;12(11):1445. doi: 10.3390/ani12111445 (PMC9179378; doi:10.3390/ani12111445)
Supplement: Supplementary file 1 [file animals-12-01445-s001.zip › animals-1672575-supplementary.pdf]

animals

MDPI

Supplementary Materials

Immunocyte Populations Observed from Birth to Weaning in Blood, Spleen and Mesenteric Lymph Nodes of Piglets

Tomoko Harayama [1], Takamitsu Tsukahara [1,2,*], Kikuto Fukuta [3], Machi Oda [4] and Ryo Inoue [1,4]

[1] Laboratory of Animal Science, Division of Applied Life Sciences, Graduate School of Life and Environmental Sciences, Kyoto Prefectural University, Kyoto 606-8522, Japan; reeforth73finag@aol.com (T.H.); ryo.inoue@setsunan.ac.jp (R.I.)
[2] Kyoto Institute of Nutrition & Pathology, Kyoto 610-0231, Japan
[3] Technical Center, Toyohashi Feed Mills, Aichi 441-1346, Japan; k-hukuta@toyohashi-shiryo.co.jp
[4] Laboratory of Animal Science, Department of Applied Biological Sciences, Faculty of Agriculture, Setsunan University, Hirakata, Osaka 573-0101, Japan; machi.oda@edu.setsunan.ac.jp
* Correspondence: tsukahara@kyoto-inp.co.jp; Tel.: +81-(0)-7-74997331

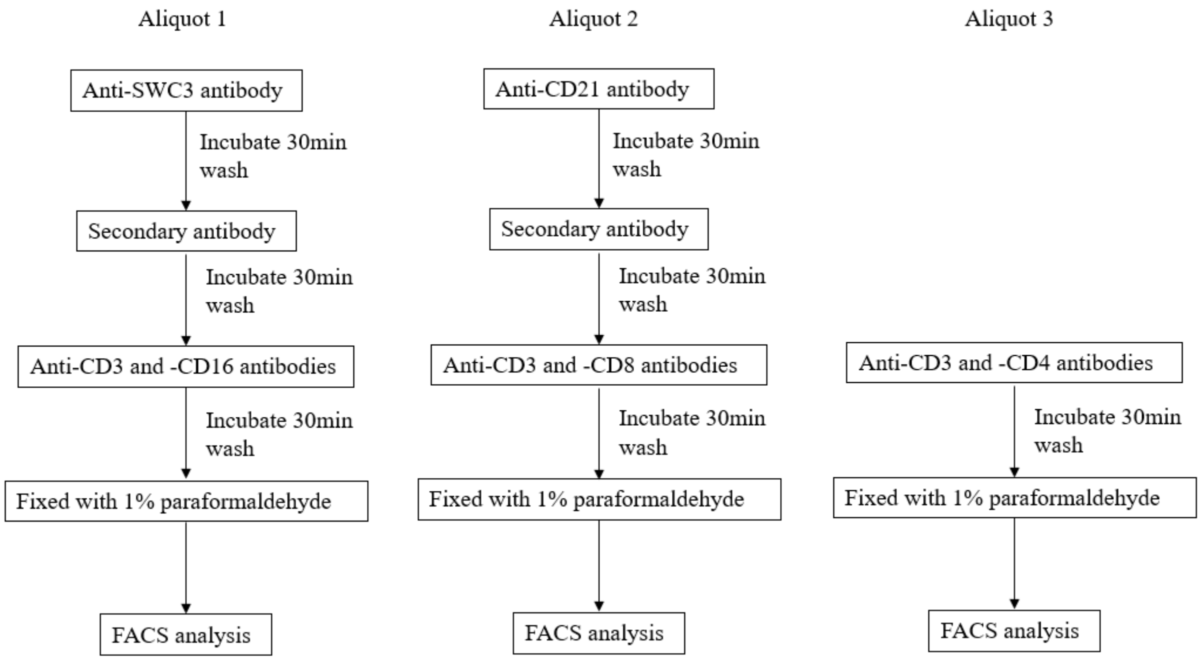

Figure S1. Flow of multiple antibody staining.

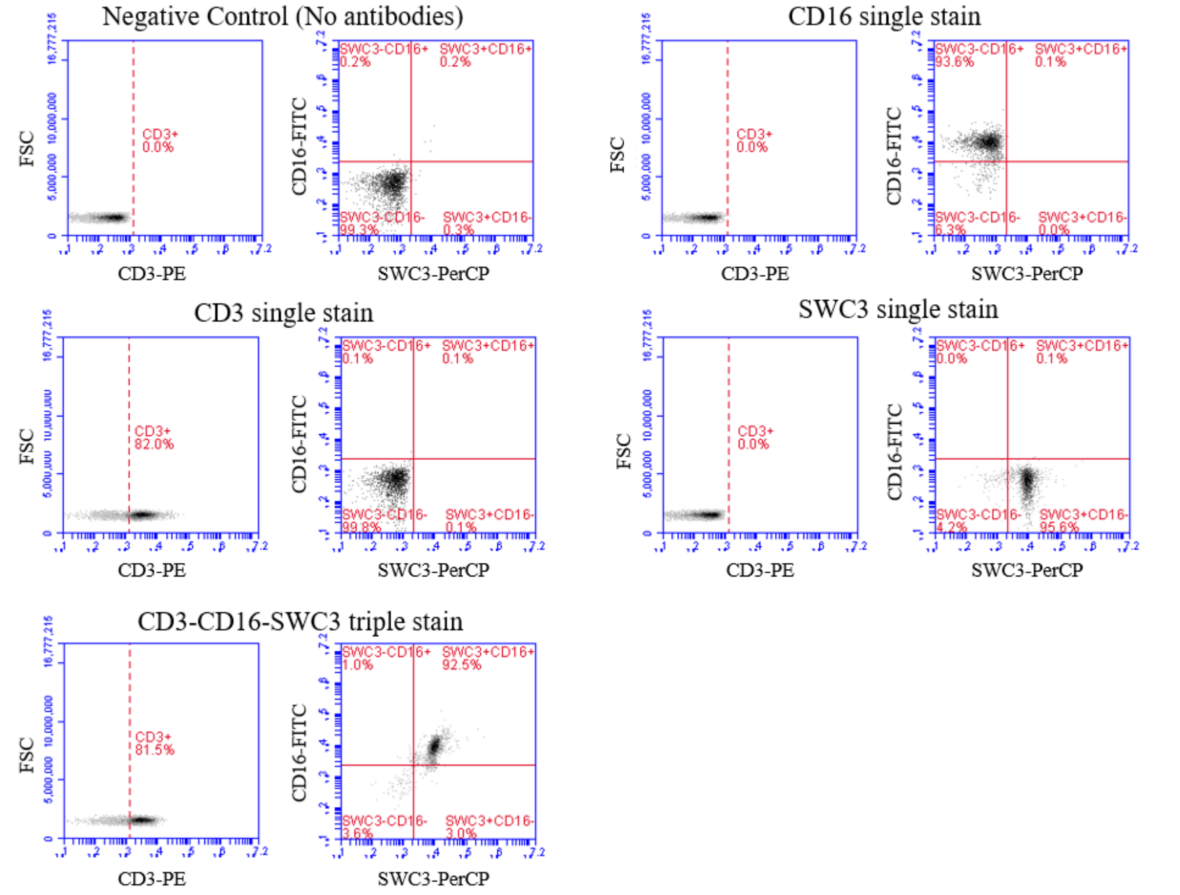

Figure S2. Effect of staining with multiple antibodies on each cell population. PBMCs were isolated from heparinized blood collected from sow and stained with either of antibodies used in this study (anti-CD3, CD16 or SWC3) or with three antibodies (anti-CD3, CD16 and SWC3) as described in materials and methods. Left panel of each staining result was dot plot gated on lymphocytes and right panel was on monocyte and granulocytes. Staining with multiple antibodies showed negligible difference in percentage of each cell population in comparison to single antibody stain. For example, percentage of SWC3+ cells was 95.7% when stained with single anti-SWC3 antibody, while it was 95.5% stained with three antibodies.

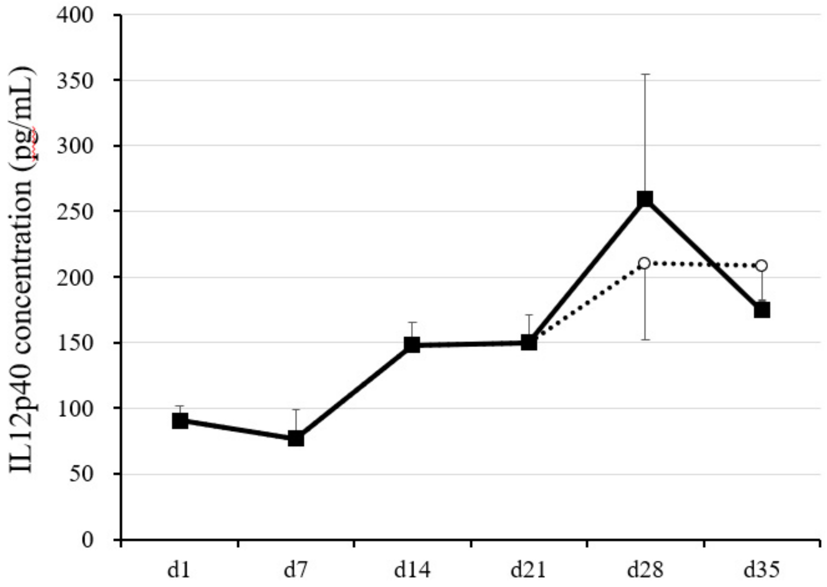

Figure S3. Concentrations of IL-12p40 in the sera of piglets. Closed squares indicate the mean values for suckling piglets. Open circles indicate the mean values for piglets weaned at 21 days of age. The error bars represent the standard errors. No statistical difference was observed between the groups.
